# Fractal-Percolation Structure Architectonics in Sol-Gel Synthesis

**DOI:** 10.3390/ijms221910521

**Published:** 2021-09-29

**Authors:** Irina Kononova, Pavel Kononov, Vyacheslav Moshnikov, Sergey Ignat’ev

**Affiliations:** 1 Department of Micro- and Nanoelectronics, Faculty of Electronics, Saint-Petersburg Electrotechnical University “LETI”, 5, pr. Popova, 197376 Saint-Petersburg, Russia; iegrachova@mail.ru (I.K.); vamoshnikov@mail.ru (V.M.); 2 Department of Descriptive Geometry and Graphics, Faculty of Basic and Human Sciences, Saint-Petersburg Mining University, 2, 21st Line, 199106 Saint-Petersburg, Russia; ignatev_sa@pers.spmi.ru

**Keywords:** sensors materials, self-assembly, metal oxide, thin films, architectonics, porous structure, fractals

## Abstract

It was developed a new technique to assess micro- and mesopores with sizes below a few nanometers. The porous materials with hierarchical fractal-percolation structure were obtained with the sol-gel method. The tetraethoxysilane hydrolysis and polycondensation reactions were performed in the presence of salts as the sources of metal oxides. The porous materials were obtained under spinodal decomposition conditions during application of the polymer sol to the substrate surface and thermal treatment of the structures. The model is based on an enhanced Kepler net of the 4612 type with hexagonal cells filled with a quasi-two-dimensional projection of the Jullien fractal after the 2nd iteration. The materials obtained with the sol-gel method were studied using the atomic force microscopy, electron microscopy, thermal desorption, as well as an AutoCAD 2022 computer simulation of the percolation transition in a two-component system using the proposed multimodal model. Based on the results obtained, a new method was suggested to assess micro- and mesopores with sizes below a few nanometrs, which cannot be analyzed using the atomic force microscopy and electron microscopy.

## 1. Introduction

Materials with a hierarchical structure [1,2,3], i.e., with a special material (substance) organization in which subunits possessing nano [4,5,6,7]- or microscopic properties are integrated into large-sized associations with a more complex new spatial structure [8,9,10] and functional capabilities, are of great relevance [11,12].

Materials obtained with architectonic [1,2,3] synthesis open great opportunities [13,14,15] in energy, environmental protection, bioengineering, catalysis, sensorics, creation of lithium-ion batteries, biomedicine, etc [16,17,18,19,20].

In [2], some methods for the synthesis of hierarchically porous structures with various chemical compositions (dual porosities: micro–micropores, micro–mesopores, micro–macropores, meso–mesopores, meso–macropores, multiple porosities: micro–meso–macropores and meso–meso–macropores).

It should be noted that according to the IUPAC recommendation three main types of pores are distinguished, i.e., micropores with the sizes below 2 nm, mesopores in the size range from 2 to 50 nm, and macropores with the sizes over 50 nm [21,22]. This classification is based on the differences in the basic mechanisms of sorption processes that take place in pores of different sizes.

Useful functions of multilevel hierarchical nanomaterials [23,24] are determined by not only a nanolevel but also the other structural levels. A structural hierarchy means a composite nature of more complex levels in comparison with the modules that form the higher level. As a result, when linked into a structure, the modules transfer some of their functions and degrees of freedom to it. At the same time, many properties of more complex structures cannot be reduced to the properties of their modules [25,26].

It was shown a variety of applications of hierarchically structured composites in catalysis, energy storage, usage and conversion, removal of pollutants, sensors, biomaterials, smart soaps, and structuring of consumer products [3].

It is known that hierarchically structured porous materials have shown their great potential for energy storage applications owing to their large accessible space, high surface area, low density, excellent accommodation capability with volume and thermal variation, variable chemical compositions and well controlled and interconnected hierarchical porosity at different length scales. Porous hierarchy benefits electron and ion transport, and mass diffusion and exchange [27].

Hierarchically porous metal-organic frameworks are versatile nanostructured materials that find applications in different fields, spanning from separation, adsorption, sensing and catalysis to health care and nanomedicine. It was shown novel opportunities for the use of functional carbohydrates as bio-based adsorbents and chemical scavengers [28].

Hierarchically porous materials with large pores in the micrometer range and small pores in the nanometer range, where the large pores facilitate mass transport and the small pores supply numerous active sites, show superiority to materials with unimodal pores in the fields of separation and adsorption [29,30].

This research focuses on the sol-gel synthesis [31,32] and diagnostics of porous materials with hierarchical fractal-percolation structure as well as the development of a new technique to assess micro- and mesopores with sizes below 10 nm, which cannot be analyzed using the AFM.

## 2. Results

### 2.1. Porous Materials with a Hierarchical Fractal-Percolation Structure Obtained Using the Sol-Gel Synthesis

Precursors used to produce the sols were easily hydrolysable compounds that form polymolecules or polysolvated complexes when interacting with water. Tetraethoxysilane (TEOS) Si(OC_2_H_5_)_4_ (Si(OR)_4_), readily soluble in many organic solvents and which main property is tendency to hydrolysis, is often used for silicon dioxide-based thin films. It is possible to perform TEOS hydrolysis and polycondensation reactions in the presence of salts as the sources of metal oxides, which significantly expands the possibilities of the method to produce multicomponent silicon dioxide-based oxide materials.

**Figure 1:**
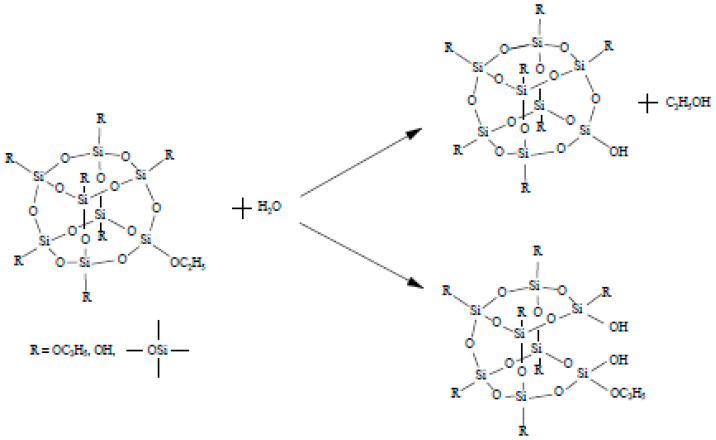
A possible hydrolysis reaction.

**Figure 2:**
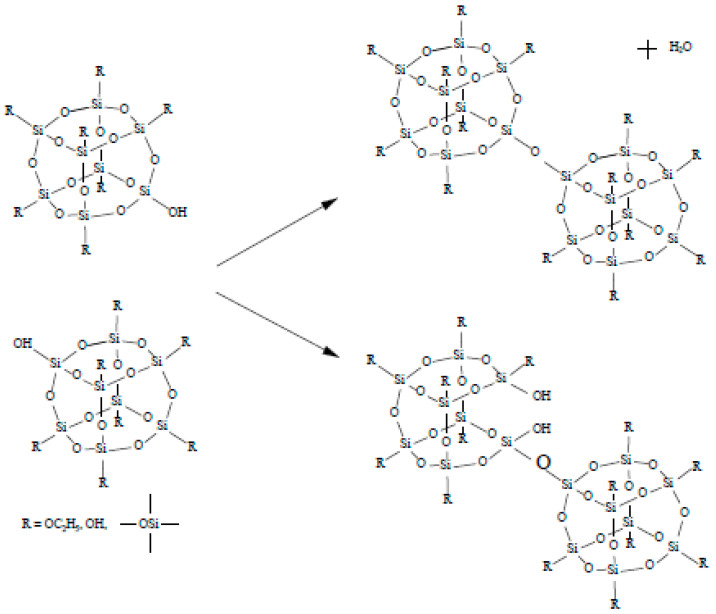
A possible polycondensation reaction.

The cyclic compounds can be obtained by using the hydrolysis process (Figure 1). Then the polycondensation reaction will progress as shown in Figure 2.

**Figure 3:**
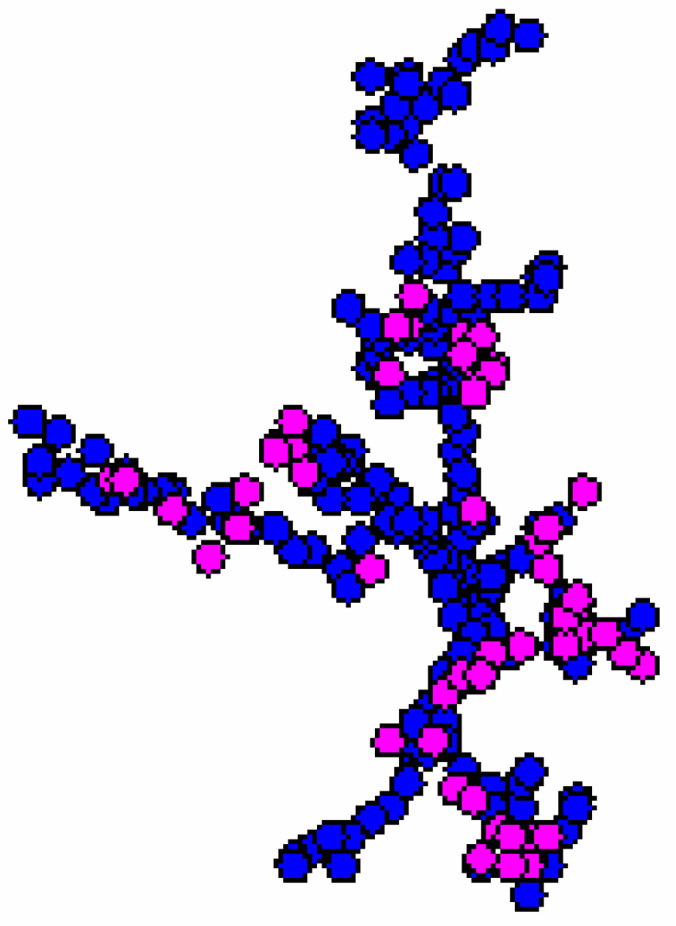
Computer simulation result within the diffusion-limited aggregation model.

**Figure 4:**
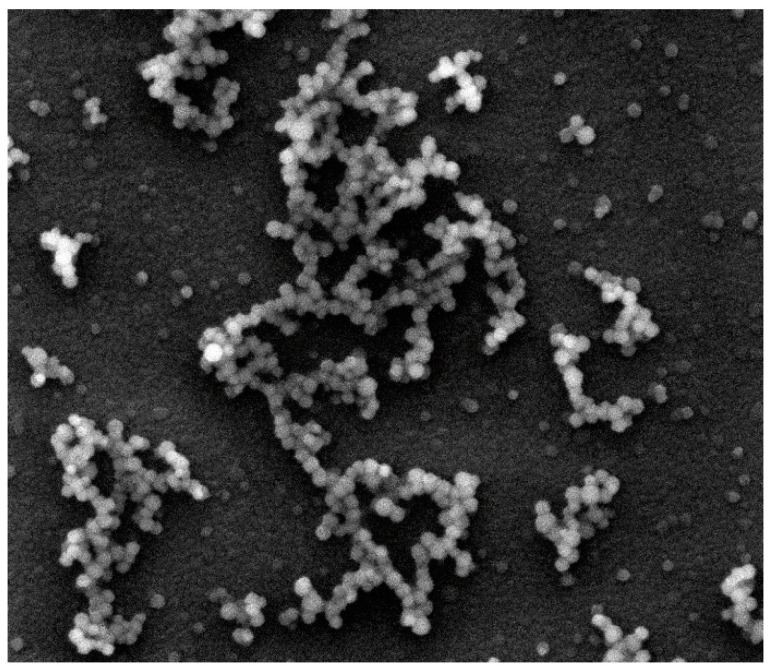
A scanning electronic microscopy image of a silicon dioxide sample illustrating the process of diffusion-limited aggregation that proceeds simultaneously with the cluster-cluster aggregation (image size 40 μm × 40 μm).

During polymerization of (Si, O)-chains a three-dimensional core (cristobalite type) is formed and a part of chains (peripheral structures) remains on the surface of the spherical particle. The growth starts in the sol with the material collection in the form of a fractal cluster of the sol particles (globules) following the DLA Model (diffusion-limited cluster-particle aggregation, Figure 3). It should be noted that according to the Witten and Sander model, aggregation phenomena take place locally as the result of suppression of the long-range repulsive forces between the sol particles, which move chaotically in the solution. This causes the aggregate to grow by joining another particle to the aggregate due to collision. Having collided with the aggregate, the particle gets attached to it at the collision site (Figure 4).

**Figure 5:**
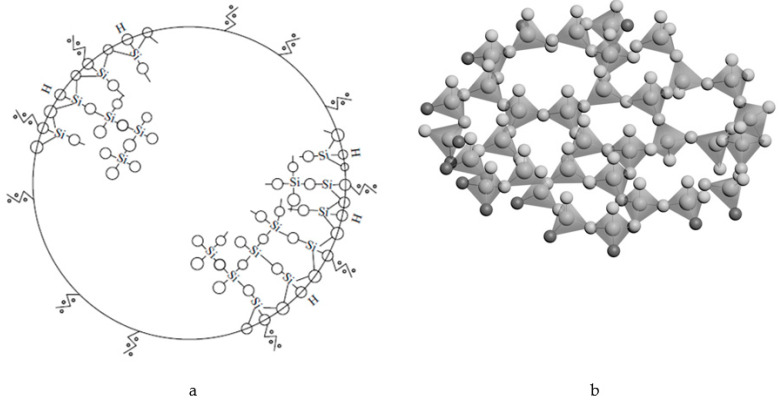
A schematic representation of the primary sol particle (**a**), united polymorphoids (**b**).

Note that the structure of the sol particle (spherical globules in Figure 3 and Figure 4) is a SiO_2_ glass-forming net (united polymorphoids). Figure 5 shows a schematic representation of the sol’s primary particle. The primary structural unit of the SiO_2_ glass-forming net is a SiO_4_ -tetrahedron where a silicon atom with the orbital radius of 107 pm and sp3 hybridization is bound to four oxygen atoms with the orbital radius of 45 pm, while the secondary structural unit is a polymorphoid of n-membered ring, i.e., a ring of SiO_4_ -tetrahedra with n Si-O-bonds or a fragment of polymorphic modification structure.

For example, with a globule radius of 1 nm, which is a glass-forming net of silicon dioxide in the form of united polymorphoids, the globule contains about 12-14 SiO_4_ –tetrahedrons (Figure 5b).

The initial sols are non-equilibrium. As the time of the practically irreversible polycondensation reactions increases (increased sol holding time), the fractal aggregates are growing (Figure 4), the number of possible permutations of particles when they are fixed on the fractal frame is decreasing (the average molecular weight of the polymerizing substances in the sol is increasing), the sol’ viscosity is rising, and the chemical cross-linking is taking place between the branched macromolecules. The process of diffusion-limited aggregation proceeds simultaneously with cluster-cluster aggregation (Figure 4).

As the sol holding time increases, there takes place disintegration into pure solvent and concentrated gel, which corresponds to expulsion of the solvent from the structural net (syneresis). Two types of syneresis are distinguished: spontaneous that can last for several years and forced, which is caused by external factors (e.g., temperature). In forced syneresis, the systems reach a balanced state over a long period of time, since almost any rearrangement of the polymer structure is associated with a change in the chain conformation, chain folding or unfolding, displacement of the chains or their parts relative to each other. All these processes require time, and the more extended sections of the chains are involved in the rearrangement the more time is needed. 

**Figure 6:**
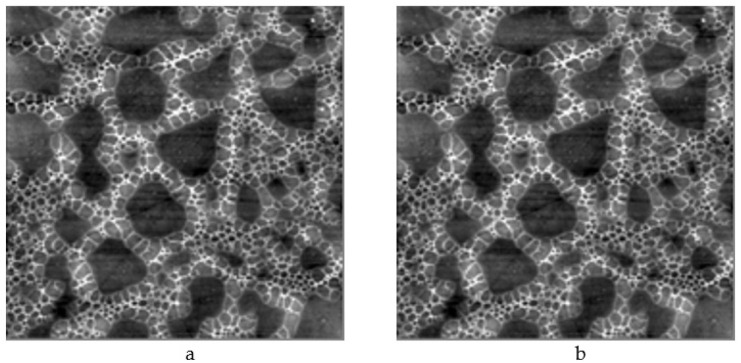
Atomic-force microscopy images of hierarchical porous nano-composites: (**a**) a two-component system of silicon and tin oxides (image size 10 μm × 10 μm); (**b**) a three-component system of silicon, iron and erbium oxides (image size 50 μm × 50 μm).

The work considers forced syneresis. Porous structures were formed by spinodal decomposition during application of the polymer sol to the substrate surface and thermal treatment of the structures, which was accompanied by “collapsing” of fractal aggregates, formation of metal oxides from inorganic salts; intensive release of volatile components accompanied by significant weight loss; compaction of the films; improved adhesion of the films to the substrate surface; increased mechanical and chemical strength of the films; transition of gels into xerogels (thin glass-like layers) and formation of porous nanomaterials (Figure 6) with multimodal pore distribution (micro-meso-macroporous structure) under spinodal decomposition conditions. 

It should be noted that the results of earlier studies (the difference by several orders of magnitude in the values of surface area calculated using two different methods. i.e., computed from experimental sorbometry data and by processing atomic force microscopy images) revealed that there exists a system of micropores with sizes below 10 nm, which cannot be detected by atomic force microscopy. Thus, the conducting branches of macroporous objects are not solid, as a rule, this is a material with a hierarchical structure (Figure 6).

### 2.2. Development of a New Method to Assess Micro- and Mesopores with Sizes below 10 nm 

**Figure 7:**
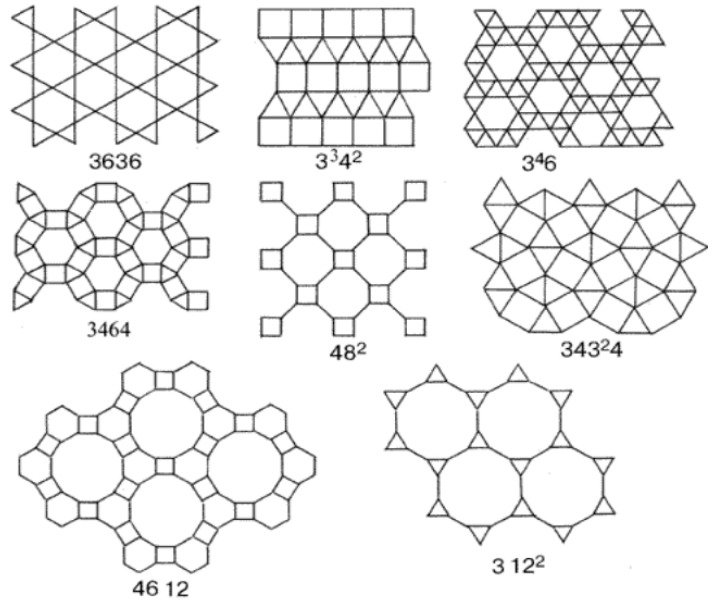
11 Kepler nets.

The paper develops a model of multimodal pore distribution, which is based on one of the 11 Kepler nets (Figure 7) with Schläfli symbol No. 4612 [33].

**Figure 8:**
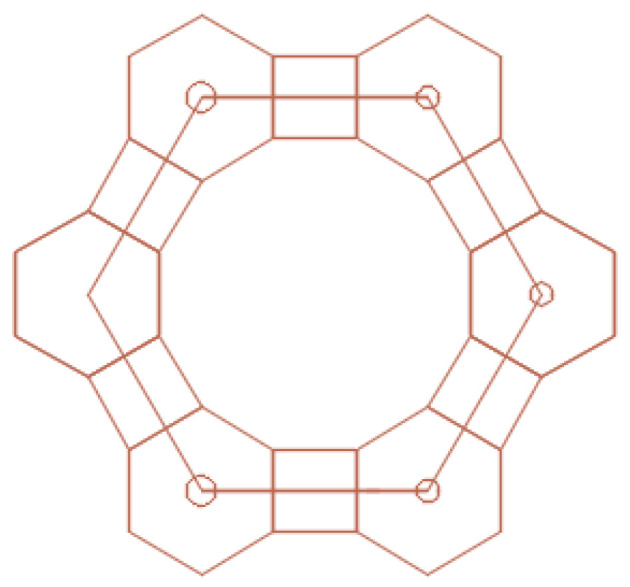
A fragment of Kepler net No. 4612 with marked centers of the regular polygons.

The Kepler nets consist of regular polygons, not necessarily of the same kind. Regular polygons (triangles, squares, hexagons, octagons, dodecagons) and their combinations fill in the flat space without gaps and overlapping. The Schläfli symbols indicate the number and type of regular polygons converging at each node of the net. The Arabic numbers correspond to the following shapes: 3 to a triangle, 4 to a square, 6 to a hexagon, 12 to a dodecagon. The top number indicates the number of polygons converging on the same node and touching each other by their sides. Number 4612 means that the net node is a vertex of one triangle, one hexagon, and one dodecagon. A square in the Kepler net with the Schläfli symbol 4612 (Figure 7) was replaced in the article by a regular hexagon with the side equal to the side of the square (Figure 8).

**Figure 9:**
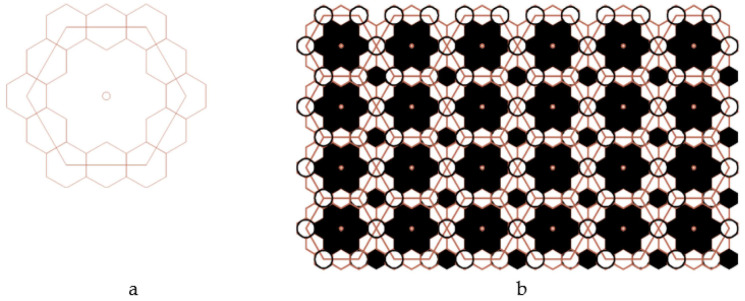
A fragment (**a**) and the enhanced Kepler net of the 4612 type (**b**).

Thus, we obtained a large hexagon, the vertices of which pass through 12 regular small hexagons, and the side of which is equal to the quadruple radius of a circle inscribed into a small regular hexagon (Figure 9a). Modeling was performed in the AutoCAD 2022 2D and 3D computer-aided design and drawing software application developed by Autodesk (Figure 9b).

**Figure 10:**
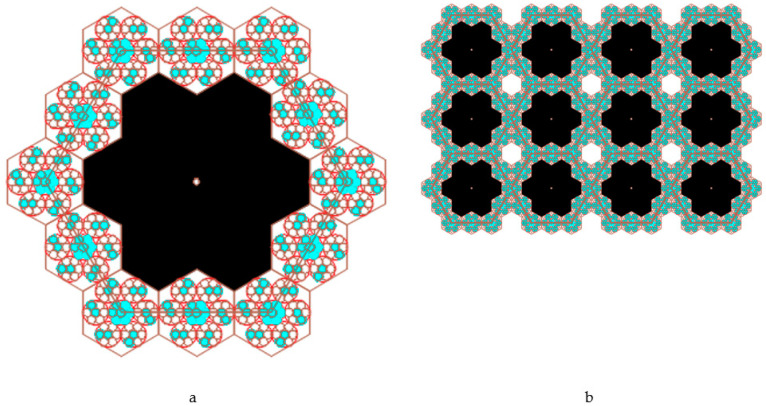
A fragment (**a**) and the enhanced Kepler net of the 4612 type (**b**) with the cells filled with a quasi-two-dimensional projection of the Jullien fractal.

Each regular hexagon in the enhanced Kepler net with Schläfli 4612 symbol was filled a quasi-two-dimensional projection of a 3D enhanced non-deterministic Jullien fractal [34] after the second iteration (Figure 10).

We limited our model to only two iteration levels (if necessary it is possible to increase the next levels as well), but already at these levels there appears such a concept as pores of different sizes.

It should be noted that a quasi-two-dimensional projection of the 3D deterministic Jullien fractal aggregate is characterized by the fractal dimensions of 1.771, while the fractal dimensions of the three-dimensional deterministic Jullien fractal aggregate is 2.335.

## 3. Discussion

**Figure 11:**
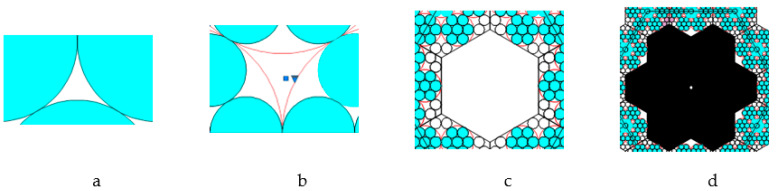
Four pore types: (**a**) type one; (**b**) type two; (**c**) type three; (**d**) type four.

The following four pore types were observed in the enhanced Kepler net of the 4612 type with cells filled with a quasi-two-dimensional projection of the Jullien fractal (Figure 11):

1. pores in the shape of a curved equilateral triangle formed by the gaps between the three touching globules with the circle radius α;
2. pores in the shape of a curved hexagon, formed by the gaps between six touching globules with the circle radius α;
3. pores formed by the gaps between the six regular hexagons filled with quasi-two-dimensional projections of the Jullien fractal after the 2nd iteration and built-up globules in the contact points of the regular hexagons;
4. pores formed by the gaps between the twelve regular hexagons.

**Figure 12:**
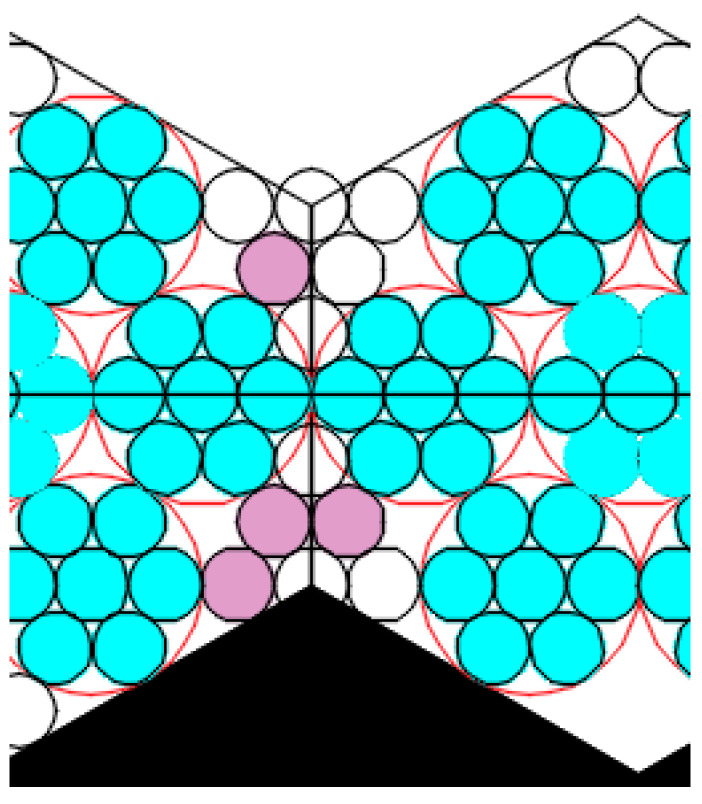
An illustration of deformation compaction of the structure at the boundary of two small regular hexagons.

The model was built based on the assumptions that a deformation compaction of the structure takes place at the boundary of two small regular hexagons (Figure 12). The model were built based on the assumptions that a deformation compaction of the structure takes place at the boundary of two small regular hexagons (Figure 12).

The small regular hexagons in the boundary zone will have a certain impact on each other, and the growth of individual hexagons will be affected according to Jullien’s law. Only those areas that are not yet organized as hexagons can be completed. The boundary between the hexagons is capable of being sufficiently stable, i.e., being in contact not by single spheres only, but by the side of the hexagon, thereby distributing the load.

Thus, it becomes possible to distinguish pores of quite different functional significance in this general pattern. Pores of the fourth type in the multimodal porous materials produced by the sol-gel method correspond to the model where some materials produce resultant products and these products leave the substance. Pores of the third type can serve other purposes, such as being microreactors where formation of some substances takes place. Pores of the first and second type act as adsorption centers, changing their electrophysical properties and redistributing electron density at the Debye shielding depth, e.g., for gas-sensitive structures.

**Figure 13:**
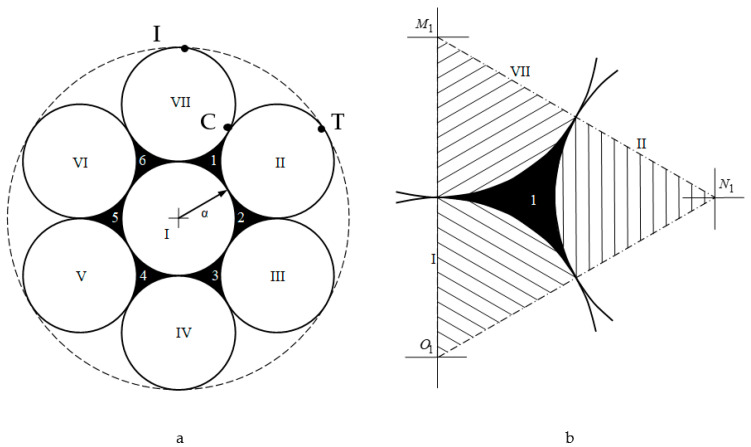
(**a**) Jullien fractal after the first iteration, (**b**) a schematic representation to calculate the area of one curved equilateral triangular cell.

The pore sizes of the first type were calculated as the area of one of the curved equilateral triangles (CETs) (marked 1-6 in black in Figure 13) formed between three joined globules with the circle radius α.

This area is equal to the difference of the area of the equilateral triangle $M_{1}N_{1}O_{1}$ (Figure 13b) with the sides 2α and vertices at the centers of the particles and the areas of the three sectors of circles with radius α (shaded in Figure 13b) with the sector angle being 60°:

(1) $$S_{CET}=\frac{{\left(2\alpha \right)}^{2}\sqrt{3}}{4}-3\pi \alpha^{2}\frac{60}{360}=\frac{\alpha^{2}\left(2\sqrt{3}-\pi \right)}{2}$$

According to the International Union of Pure and Applied Chemistry (IUPAC) recommendations, the pore size is characterized by the radius of the circle inscribed in the pore. Let us estimate the size of the first pore type (Figure 11a) as the radius of the circle inscribed into a curved equilateral triangle CET:

(2) $$r_{1}=\frac{\sqrt{S_{\mathrm{CET}}}}{3^{3/4}}=\frac{\sqrt{0.5\left(2\sqrt{3}-\pi \right)}}{3^{3/4}}\alpha$$

We define the area of the curved isosceles triangle, e.g., CIT as:

(3) $$\begin{array}{l} 6S_{CIT}=\pi {\left(3\alpha \right)}^{2}-7\pi \alpha^{2}-6\frac{\alpha^{2}\left(2\sqrt{3}-\pi \right)}{2}\\ =9\pi \alpha^{2}-7\pi \alpha^{2}-6\frac{\alpha^{2}\left(2\sqrt{3}-\pi \right)}{2}\\ S_{CIT}=\frac{\pi \alpha^{2}}{3}-\frac{\alpha^{2}\left(2\sqrt{3}-\pi \right)}{2} \end{array}$$

**Figure 14:**
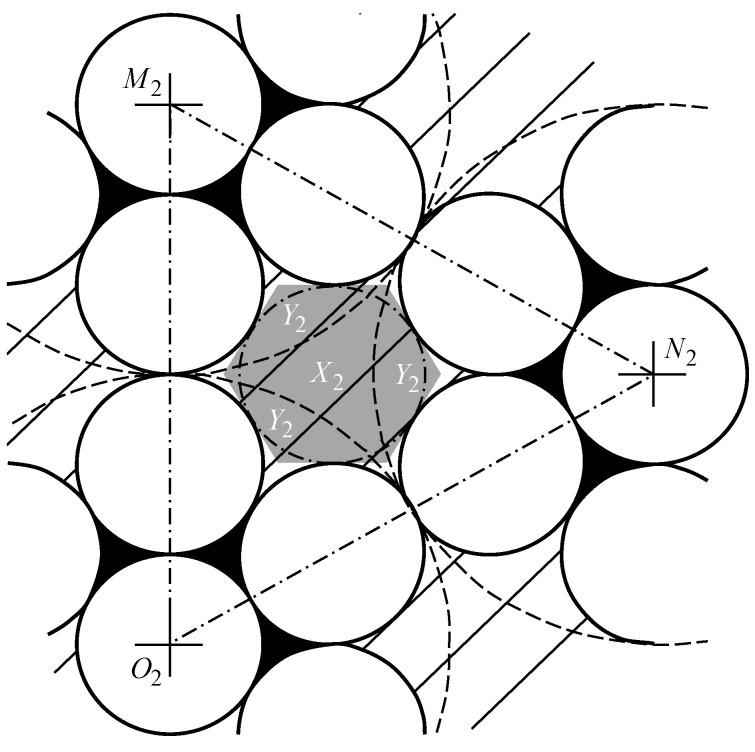
The area calculation of the curved triangle $X_{2}$.

The area of pores of the second type in the form of curved hexagon (CH) $S_{CH}$ (shown in Figure 14 as a striped zone) is equal to the total area of the curved equilateral triangle $X_{2}$ and the three identical curved isosceles triangle $Y_{2}$, that have the area of $S_{CIT}$.

The area of the curved triangle $X_{2}$ is equal to the difference of the area of the equilateral triangle $M_{2}N_{2}O_{2}$ (Figure 14) with the side of 6α and the sum of areas of three 60-degree sectors bounded by arcs of radius circles 3α:

(4) $$S_{X_{2}}=\frac{{\left(6\alpha \right)}^{2}\sqrt{3}}{4}-3\pi {\left(3\alpha \right)}^{2}\frac{60}{360}$$

The area of the curved isosceles triangle $Y_{2}$ is equal to 1/6 of the difference in the area of the circle of radius 3α, circumscribed around the Jullien unit formed after the first iteration and the sum of the areas of seven identical spherical particles of radius α $\left(7\alpha^{2}\right)$ and the six pores of the Jullien unit after the first iteration:

(5) $$S_{Y_{2}}=\frac{\pi {\left(3\alpha \right)}^{2}-7\pi \alpha^{2}-6S_{CET}}{6}$$

Then from Equations (4) and (5) it was found:

(6) $$\begin{array}{c} S_{CH}=S_{X_{2}}+3S_{Y_{2}}=9\alpha^{2}\frac{2\sqrt{3}-\pi }{2}+3\frac{2\pi \alpha^{2}-6S_{CET}}{6}\\ =9S_{CET}+\pi \alpha^{2}-3S_{CET}=\pi \alpha^{2}+6S_{CET}.\\ S_{CH}=\pi \alpha^{2}+6S_{CET} \end{array}$$

Let us estimate the size of the second type of pores (Figure 11b) after the second iteration as the radius of the circle, inscribed into the regular hexagon of the pore (Figure 14, marked in dark color). Given the definition of this radius

(7) $$r_{2}=\frac{\sqrt{3}}{2}a$$

where *a* is the side of the regular hexagon and the definition of this hexagon’s area: a then the radius of the second type of pores is

(8) $$r_{2}=\frac{1}{3^{1/4}}\frac{\sqrt{2S_{\mathrm{CH}}}}{2}$$

Estimation of radius of the third type of pores was made by means of AutoCAD 2022 package, for this purpose we graphically defined the side of the regular hexagon (Figure 11c) as $10a+\frac{a}{2.54966}$. Then

(9) $$r_{3}=\frac{\sqrt{3}}{2}\left(10a+\frac{a}{2.54966}\right)$$

The pore radius of the fourth type was defined as the radius of the circle inscribed in the regular hexagon which is inscribed in the fourth type pore of irregular shape (Figure 11d) with accounting for the graphical definition of the side of the regular hexagon 20.78415a. Then

(10) $$r_{4}=\frac{\sqrt{3}}{2}20.78415\alpha$$

**Figure 15:**
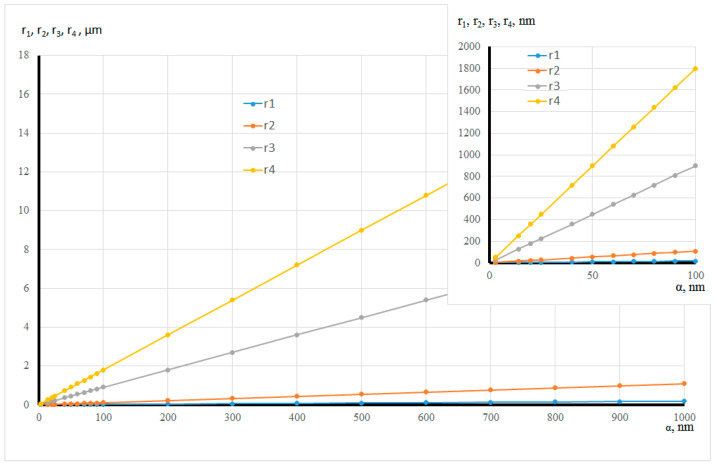
Dependence of the radius size of the four pore types on the globule radius.

Figure 15 and Table 1 present the estimation of the radii of the four pore types versus the globule radius. The values show that a micro-mesoporous system is formed with the globule radius of 1 nm. Macropores are observed when the globule radius is 2.78 nm, and then a micro-meso-macroporous system is formed.

The paper considered formation of a percolation spanning cluster on an enhanced Kepler net of the 4612 type with the cells filled with a quasi-two-dimensional projection of the Jullien fractal. Nanocomposites consisted of two types of the unit cells (globules of the same size), being conductors (Type 1 cells) and ideal dielectrics (Type 2 cells). When the proportion of the Type 1 cells in the material increases against the Type 2 cells, the electrical properties of the system change from fully insulating to fully conducting. With small concentrations of the conductive cells, they are isolated from each other and from the electrodes by the dielectric cells. At a certain proportion, called the percolation threshold, a spanning cluster emerges, i.e., conductivity takes place. 

The calculation of the percolation threshold was performed in AutoCAD 2022, a fully functional professional computer graphics program.

The vertical flow was considered, i.e., the electrodes were assumed to be applied to the upper and lower parts of the structure. The value of the percolation threshold was taken as the ratio of the number of conducting cells to the total number of cells. The estimation produced the value of 0.68. It should be noted that this value is lower than the percolation threshold value for the two-dimensional lattice with square unit cells (0.59 for node setting) and higher than the percolation threshold value for the two-dimensional lattice with honeycomb type unit cells (0.7 for node setting).

**Figure 16:**
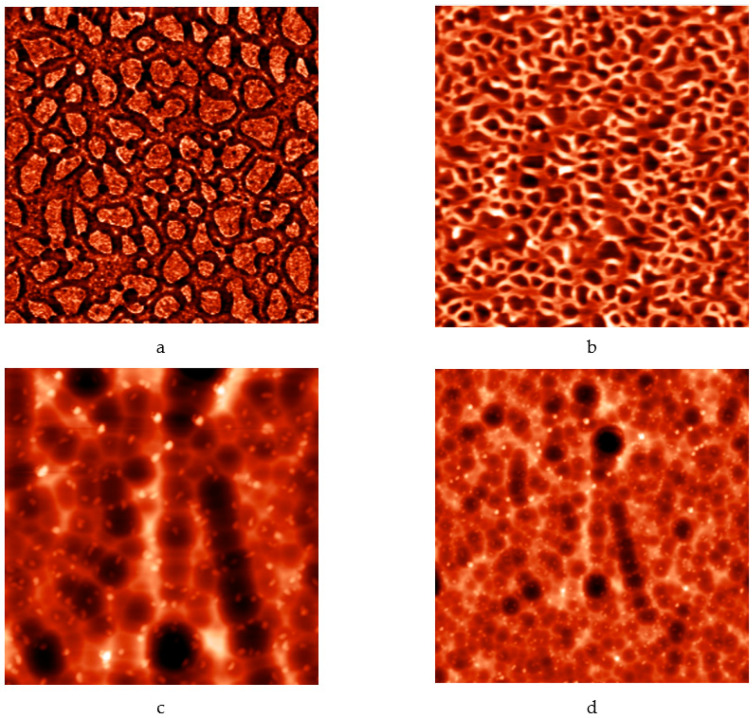
Atomic-force microscopy images of porous structures based on tin dioxide and silicon: (**a**) before etching in hydrofluoric acid (image size 5 μm × 5 μm); (**b**) before etching in hydrofluoric acid (image size 8 μm × 8 μm); (**c**) after etching (image size 20 μm × 20 μm); (**d**) after etching (image size 40 μm × 40 μm).

It is worth mentioning that the model of the enhanced Kepler net of the 4612 type with hexagonal cells filled with a quasi-two-dimensional projection of the Jullien fractal after the 2nd iteration chosen in this work is confirmed by the results of etching of porous structures based on tin dioxide and silicon in hydrofluoric acid. Formation of hexagons was observed after etching (Figure 16).

**Figure 17:**
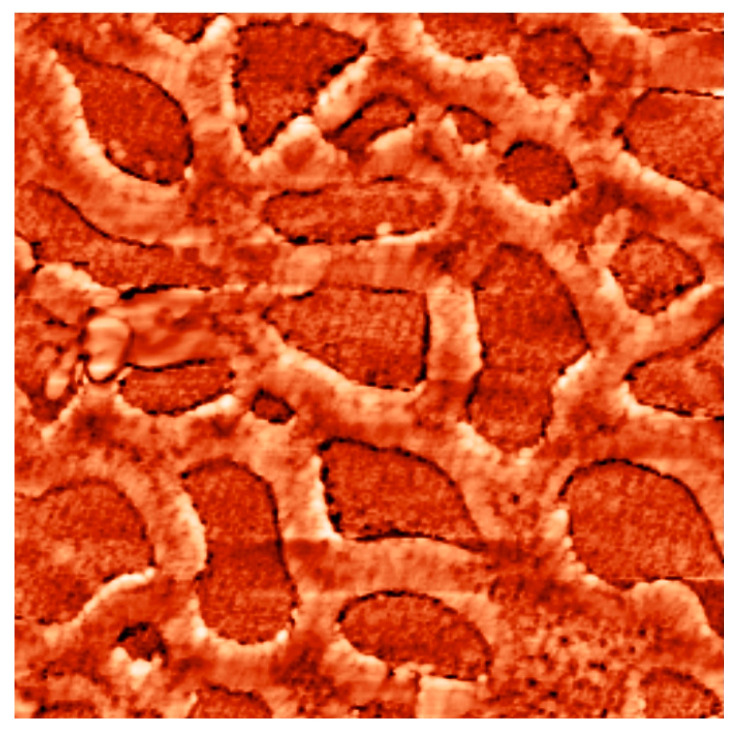
Atomic-force microscopy image (image size 2 μm × 2 μm) of porous structure based on tin dioxide and silicon dioxide with two types of pore sizes 200 nm and 400 nm.

The study tested the adequacy of the proposed model. The conductive necks between the third and fourth types of pores were estimated as 7 to 22a. This means that with the necks of 180–500 nm the globule radius will be around 25 nm, then according to the calculated data the sizes of the third and fourth pore types (visible in atomic force microscopy) will correspond (see Table 1) to 225 nm and 450 nm. Figure 17 shows a tin dioxide-based porous structure with the pore sizes of two types 200 nm and 400 nm. The sizes of the second pore type in the model for a=25 nm is about 27 nm, the atomic force microscopy confirms that pores with the sizes of 10–20 nm are observed in the same structure. The sizes of around 4 nm of the first type of pores were not observed in the experimental samples in the model due to the limitations of the AFM probe radius. Thus, the appropriateness of the model is confirmed (Figure 17).

Thereby, the paper proposes a method to assess the pores of the first and second type, knowing the experimental sizes of pores of the third and fourth types.

1. To estimate the size of pores of the third and fourth type in a multimodal model based on an enhanced Kepler net of the 4612 type with hexagonal cells filled with a quasi-two-dimensional projection of the Jullien fractal using atomic force microscopy (AFM).
2. To estimate the experimental size of the necks between the third and fourth types of pores based on the AFM data.
3. To calculate the radius size of the globule representing the glass-forming net of silicon dioxide by relating the experimental neck size to 7 and to 11.
4. To calculate the size of the first and second types of pores using the ratios (from Equations (1), (2), (6) and (8)):
  $$r_{1}=\frac{\sqrt{0.5\left(2\sqrt{3}-\pi \right)}}{3^{3/4}}\alpha .$$
  $$r_{2}=\frac{1}{3^{1/4}}\frac{\sqrt{2\left(\pi \alpha^{2}+3\alpha^{2}\left(2\sqrt{3}-\pi \right)\right)}}{2}$$

Estimate the number of SiO_4_ -tetrahedrons in the SiO_2_ glass-forming net, knowing the globule radius and the neck size.

## 4. Materials and Methods

**Figure 18:**
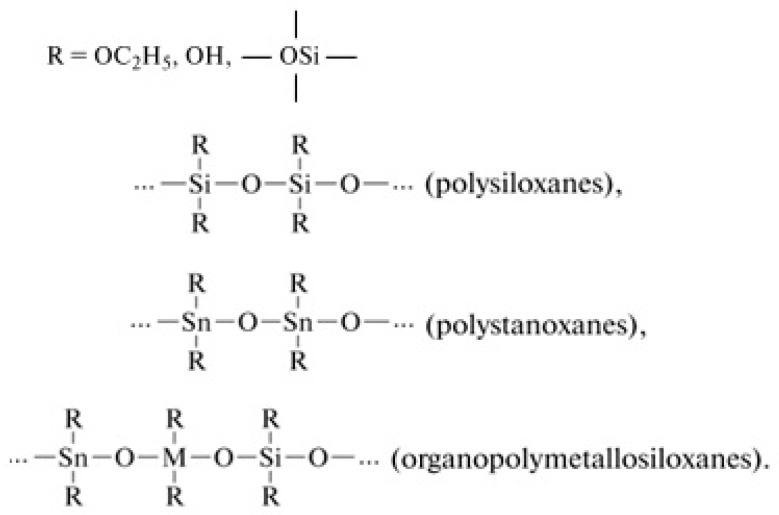
Examples of organoelement compounds.

Gas-sensitive sensor materials of two- or three-component systems based on silicon dioxide and metal oxides (tin, iron, erbium) produced by the sol-gel method were investigated in this study. The tetraethoxysilane (the ethylester of orthosilicic acid (TEOS), Si(OC_2_H_5_), ТУ 2637-187-44493179-2014, Russia) hydrolysis and polycondensation reactions were performed in the presence of salts (SnCl_2_⋅2H_2_O (GOST 36-78, Russia), FeCl_3_⋅6H_2_O (GOST 4147-74, Russia), Er(NO_3_)_3_⋅5H_2_O (ТУ 6-09-4676-78, Russia)) as the sources of metal oxides (SnO_2_, Fe_2_O_3_, Er_2_O_3_), which substantially extends the possibilities of the preparation of silica-containing multicomponent oxide materials. Note that, in addition to the formation of polysiloxanes, the formation of other organoelement compounds is possible, for example (Figure 18), polytitanoxanes, polystanoxanes, organopolymetallosiloxanes, and others (compounds whose chains are made up of carbon atoms and a heteroatom (excepnitrogen, oxygen, and sulfur atoms)):

As solvents, we used simple alcohols (ethanol, isopropanol, and butanol). The porous materials were obtained under spinodal decomposition conditions during application of the polymer sol to the substrate surface and thermal treatment (300–900 C) of the structures. Films can be produced from sols by spin casting, which ensures a more complete alkoxide hydrolysis on the substrate surface, followed by polycondensation and the formation of spatial structures in the form of gels. Freshly prepared gels consist of gel-like products (whose inorganic network structure retains water), organic solvents, and unreacted substances. Heat treatment of the gels initiates the following processes: the formation of metal oxides from the inorganic salts; active evolution of volatile components, accompanied by a considerable weight loss; film densification; improvement of film adhesion to the substrate surface; increase in the mechanical strength and chemical stability of the films; and transformation of the gels into xerogels (thin glassy layers). Since the structural gel network retains water and organic solvents, heat treatment of the samples can be followed by the formation of porous nanomaterials under spinodal decomposition conditions.

The materials obtained with the sol-gel method were studied using the atomic force microscopy, electron microscopy.

The specific surface area of the samples was determined by temperature-programmed desorption measurements. The parameters of the porous structure were studied on a device of the SORBI series (META, Novosibirsk, Russia) by comparing the volumes of the adsorbate gas (nitrogen) sorbed by the test sample and thestandard sample of material (Al_2_O_3_) provided by META with the known specific surface area of 106 m^2^/g. The specific surface was determined in the Brunauer–Emmett–Teller (BET) polymolecular model. The external specific surface was calculated from the adsorption branch of the isotherm at four relative partial pressures of the nitrogen adsorbate gas 0.15, 0.20, 0.30, and 0.40.

The calculation of the percolation threshold was performed in AutoCAD 2022 using the properties of dynamic blocks. All the globules in the multimodal model were involved in creating the dynamic block identifier. Each globule was assigned an individual attribute that was linked to the AutoCAD table cells through the properties of the object fields, which were automatically updated when the associated values changed. The AutoCAD table cells were in turn linked to Excel tables. An array of numbers was generated in the Excel table using a random number generator.

Based on the results obtained, a new method was suggested to assess micro- and mesopores with sizes below a few nanometrs, which cannot be analyzed using the atomic force microscopy and electron microscopy.

## Figures and Tables

**Table 1:** Assessment of the radius size of the four pore types.

a,nm	$r_{1},nm$	$r_{2},nm$	$r_{3},nm$	$r_{4},nm$
1	0.18	1.09	9.00	18.00
2.7	0.48	2.94	24.30	48.60
2.8	0.49	3.05	25.20	50.40
2.9	0.51	3.16	26.10	52.20
25	4.40	27.23	225.00	449.99
50	8.81	54.46	450	899.98
